# Human Mesenchymal Stem Cells Retain Multilineage Differentiation Capacity Including Neural Marker Expression after Extended *In Vitro* Expansion

**DOI:** 10.1371/journal.pone.0137255

**Published:** 2015-09-10

**Authors:** Rachel K. Okolicsanyi, Emily T. Camilleri, Lotta E Oikari, Chieh Yu, Simon M. Cool, Andre J. van Wijnen, Lyn R. Griffiths, Larisa M. Haupt

**Affiliations:** 1 Genomics Research Centre, Institute of Health and Biomedical Innovation, Queensland University of Technology, Brisbane, QLD, Australia; 2 Department of Orthopedic Surgery & Biochemistry and Molecular Biology, Mayo Clinic, Rochester, MN, United States of America; 3 Institute of Medical Biology, Glycotherapeutics Group, A*STAR, Singapore, Singapore; University of Torino, ITALY

## Abstract

The suitability of human mesenchymal stem cells (hMSCs) in regenerative medicine relies on retention of their proliferative expansion potential in conjunction with the ability to differentiate toward multiple lineages. Successful utilisation of these cells in clinical applications linked to tissue regeneration requires consideration of biomarker expression, time in culture and donor age, as well as their ability to differentiate towards mesenchymal (bone, cartilage, fat) or non-mesenchymal (e.g., neural) lineages. To identify potential therapeutic suitability we examined hMSCs after extended expansion including morphological changes, potency (stemness) and multilineage potential. Commercially available hMSC populations were expanded *in vitro* for > 20 passages, equating to > 60 days and > 50 population doublings. Distinct growth phases (A-C) were observed during serial passaging and cells were characterised for stemness and lineage markers at representative stages (Phase A: P+5, approximately 13 days in culture; Phase B: P+7, approximately 20 days in culture; and Phase C: P+13, approximately 43 days in culture). Cell surface markers, stem cell markers and lineage-specific markers were characterised by FACS, ICC and Q-PCR revealing MSCs maintained their multilineage potential, including neural lineages throughout expansion. Co-expression of multiple lineage markers along with continued CD45 expression in MSCs did not affect completion of osteogenic and adipogenic specification or the formation of neurospheres. Improved standardised isolation and characterisation of MSCs may facilitate the identification of biomarkers to improve therapeutic efficacy to ensure increased reproducibility and routine production of MSCs for therapeutic applications including neural repair.

## Introduction

Embryonic (pluripotent) and adult stem cells (multipotent) represent a biological reservoir of cells that retain differentiative ability into a number of cell types to accommodate tissue homeostasis and repair. Traditionally, adult mesenchymal stem cells (MSCs) have been isolated from the bone marrow (an invasive procedure) but other sources including fat, umbilical cord blood, dental pulp, skeletal muscle and amniotic fluid are clinically relevant alternatives [1–7]. The multilineage potential of MSCs, their relative ease of isolation and culture, as well as their high *ex vivo* expansive potential makes these cells an attractive therapeutic tool [8–10]. However, MSCs do not have unlimited proliferative capacity and their ability to differentiate into multiple lineages is influenced by multiple factors including donor age [11]. Contributing to current disadvantages for these cells in regenerative medicine is the imprecision of the identification and classification of MSCs from different biological sources and/or laboratories, with differentiative potential shown to vary dependant on the source (reviewed in [12,13]).

The standard definition according to the International Society of Cell Therapy identifies properties of MSCs, regardless of their origin and method of isolation, as: capable of adhesion to plastic, tri-lineage differentiation into adipo-, chondro- and osteocytic cells and expression of CD105, CD90, CD73 without expression of CD34, CD45, CD11 and HLA-DR [14,15]. In addition, along with the common tri-lineage of bone, cartilage and fat, MSCs have been demonstrated to retain the ability to differentiate toward neural lineages [16–19]. Most recently, MSC ability to generate ectopic bone tissue was shown to positively correlate with CFU-F efficiency, cell size and their ability for long-term growth and the expression of STRO-1, *DERMO-1* and *TWIST-1* [20].

Along with those listed above, other cell surface markers most commonly reported as positive in MSCs include STRO-1, CD166, CD146, CD106, CD105, CD90, CD73, CD54, CD44, CD34, CD29 and CD13, while the most commonly reported negative markers include CD106, CD49d, CD45, CD34, CD31, CD14, CD11b and CD10 [21,22]. A number of these markers have been reported as both positive and negative, demonstrating the accepted inconsistency observed in the cell surface profile of MSCs [22]. In addition, several of these markers are also widely expressed on non-stem cells and cancer cells, making it very difficult to distinguish MSCs from neighbouring cells *in vivo* and in tissue preparations [15,23]. This confusion is further compounded by conflicting evidence surrounding common markers such as CD45 and CD44 [22]. As such, to date, the literature has focused more closely on the commonalities of markers positively expressed by MSCs rather than any identified differences [22].

Important routine functions of MSCs are executed during tissue growth and repair, where elevated demand for precursors requires recruitment of uncommitted progenitors from other sources [9,24–28] with migrating stem cells differentiating only when they reach an appropriate microenvironment in which to flourish [29,30]. As such, the mechanisms regulating the ability of MSCs to migrate from the bone marrow to distant sites of injury, including the brain [31], are of great therapeutic interest and significance. Evidence supporting the potential of MSCs to give rise to non-mesenchymal tissues includes work by our group under standard culture conditions using commercially available MSCs [32], and by Foudah *et al* in freshly isolated bone marrow MSCs during culture and following osteogenic and adipogenic lineage differentiation [33]. In addition, after injection into neonatal mouse brains, murine MSCs have been shown to migrate throughout the forebrain and cerebellum and differentiate into astrocytes [34].

However, to more fully identify and exploit the therapeutic potential of MSCs, a comprehensive definition of stemness, lineage, cell surface markers and transcription factors, along with source, isolation and expansive potential of the cells is required. In addition, the expression by adult MSCs of non-immunogenic surface antigens (MHC class I not MHC class II) [35] provide the opportunity to transplant MSCs into an allogeneic host without the need for immunosuppression [36] making commercially isolated and characterised MSCs viable therapeutic contenders due to consistent, reproducible isolation protocols. Here, we have expanded *in vitro* commercially available human bone marrow derived MSCs (hMSCs) and monitored the cells for morphological changes along with the expression of specific markers influencing lineage specification during extended culture. The cells were characterised using commercially available panels to identify cell surface MSC markers as well as markers of neural stem cell lineages. In addition, cultures were examined by immunocytochemistry, Western blot and Q-PCR at distinct phases of growth, during osteogenic and adipogenic lineage differentiation and neurosphere formation for changes in MSC-specific and neural-specific marker expression.

To date, the majority of studies examining the neural potential of hMSCs have examined expression of neural markers at early passages after brief (before P+3) expansion *in vitro*, with only a few studies examining neural potential after extended (after P+10) culture [33,37]. Under our culture conditions, hMSCs were successfully differentiated (osteogenic and adipogenic lineages) throughout expansion (Phase A (P+5), Phase B (P+7), Phase C (P+13)) with cells maintaining neural potential at these growth phases. In addition, Phase A cultures (P+5) cells formed neurospheres demonstrating maintenance of mesenchymal and neural lineage potential during expansion and multiple passaging of hMSCs.

## Materials and Methods

### Cell Culture

Five human mesenchymal stem cell (hMSCs) populations isolated from the iliac crest of normal donors were obtained from Lonza (Australia) and expanded as a monolayer culture. These cells were obtained through informed consent (see manufacturer’s supporting documentation (USWV-10276)) and have been used previously in numerous studies [38–40]. Cultures were maintained in human mesenchymal stem cell growth media (hMSCGM) containing basal medium (hMSCBM) supplemented with 10% human mesenchymal stem cell growth supplement, 100U/mL gentamycin/ampicillin and 10% L-glutamate (Lonza, Australia). Cells were grown in a 5% CO_2_ humidified atmosphere at 37°C and plated at 3000 cells/cm^2^ in 100mm culture dishes (Corning, Australia) in maintenance media. For immunocytochemistry (ICC) experiments cells were grown in CC2 coated, 8 chamber glass slides (Labtek, Australia). For Q-PCR experiments cells were plated at 3000 cells/cm^2^ in 6 well plates (3x10^4^ per well; Corning) and RNA harvested after three days.

### hMSC Differentiation

For differentiation protocols, hMSCs were plated at 3000 cells/cm^2^ in 6 well plates (3x10^4^ per well). Cells were plated in hMSCGM and allowed to attach overnight.

#### Adipogenesis

Following plating, cells were allowed to proliferate in hMSCGM until >95% confluent, prior to contact inhibition. Phase C cultures (P+13) cells did not exceed 60% confluence and differentiation protocols were applied after 7 days in culture. When confluent, the hMSCGM was replaced with Adipogenic Induction Medium (AIM; Lonza). After three days, AIM was replaced with Adipogenic Maintenance Medium (AMM; Lonza). The cells underwent three cycles of three days in AIM followed by three days in AMM. At the end of this period, cells were maintained in AMM for a further seven days. At the completion of adipogenic induction, cells were fixed in 4% paraformaldehyde (PFA; Sigma-Aldrich, Australia) prior to staining of lipid vacuoles with Oil Red O (Sigma-Aldrich) and plates imaged on a flat-bed scanner.

#### Oil Red O Staining

Briefly, at the end of the induction/maintenance period, cells were washed twice with 1X phosphate buffered saline (PBS) and then fixed in 4% PFA for 15 min. A second wash in 1X PBS was conducted and then 70% ethanol was added to cover the monolayer. Plates were stored at 4°C until required for staining.

Oil Red O was diluted to a 1% working solution in ddH_2_O immediately prior to use. Prior to staining, cells were washed in 1X PBS. The cell monolayer was incubated in 1mL of Oil Red O working solution at room temperature for 10–20 minutes. The cells were then washed 3 times in ddH_2_O and plates scanned.

#### Osteogenesis

Following plating, cells were allowed to attach overnight. The following day, hMSCGM was replaced with Osteogenic Induction Media (OIM; Lonza). Media was changed every 2–3 days and the cells maintained in OIM for 14–21 days. At the completion of osteogenic induction the cell monolayer was fixed in 4% PFA prior to staining with Alizarin Red (Sigma-Aldrich) and von Kossa stain (Merck-Millipore, Australia) for calcium deposition. Plates were imaged on a flat-bed scanner.

#### Alizarin Red Staining

As for Oil Red O staining, cells were fixed in 4% PFA and stored in 70% ethanol at 4°C prior to staining. Alizarin Red dye powder was resuspended in ddH_2_O, filtered and diluted to a 1% stock solution in ddH_2_O and the pH adjusted to pH 4.3 with 1M sodium hydroxide (NaOH; Sigma-Aldrich). Prior to use, the stock solution was diluted 1/10 to a working solution. To each well of a 6 well plate, 2mL of diluted Alizarin Red dye was added and cells incubated at room temperature for 30 min with gentle shaking. Cells were washed, the plates air dried and visualised on a flat-bed scanner.

#### Neurosphere formation

To induce neurosphere formation, hMSCs were plated at Phase A (P+3) at ~3000 cells/m^2^ (2.5 x 10^5^ /100mm dish) in standard culture conditions until 70–80% confluent. Cells were passaged using 0.25% trypsin-EDTA (Life Technologies) to dissociate cells from culture dishes. After subsequent passaging, all Phase A (P+5) cells (3-5x10^6^) were plated in low attachment 100mm culture dishes (Corning) in Knockout DMEM/F12 media (KO DMEM/F12; Life Technologies) with 100U/mL penicillin 1μg/mL streptomycin, 20ng/mL epidermal growth factor (EGF; R&D Systems), 20ng/mL basic fibroblast growth factor (bFGF; R&D Systems) and 10μg/mL heparin (Sigma Aldrich). Cells began clustering to form spheres after a few hours and spheres were observed in increasing numbers from three hours to three days. At day three, the media was changed using a 40μm cell filter (Interpath) and fresh growth factors added to the culture media. Spheres were maintained in KO DMEM/F12 for 7 days after which they were imaged for live/dead analysis using FDA/PI (1:1000 dilution) and remaining cells harvested for RNA and protein.

Cells were harvested by removing culture medium from culture dishes washing the cell monolayer twice with 1X PBS on ice. Following washes, 1mL Trizol or 500uL protein-lysis buffer was added to the cell monolayer, the monolayer homogenised and stored at -80°C for Q-PCR and WB applications respectively. Protein-lysis buffer is described below (Western blotting).

### Immunocytochemistry

Phase A (P+5), Phase B (P+7) and Phase C (P+13) cells were plated at 12,500 cells/chamber into CC2 coated 8-chamber glass slides. Following overnight attachment cells were fixed in 4% paraformaldehyde (PFA) and stored in 70% ethanol at 4°C until staining. Cells were then blocked in either 1% normal goat serum (NGS; Sapphire Bioscience; Australia) in 1X PBS for MSC markers, 5% normal donkey serum (NDS; Sapphire Bioscience) in 1X PBS (non-permeable; for O1) or 5% NDS, 0.3% Triton X-100 in 1X PBS (permeable; for internal neural makers) for 1 hr. Primary antibodies were diluted in appropriate blocking solutions and incubated at 4°C overnight. Slides were washed three times in appropriate blocking solutions and then blocked for a further 30 min before incubation for 30 min with appropriate secondary antibodies. Secondary antibodies used were: Chromeo488 (goat anti-rabbit; Life Technologies, Australia), Chromeo546 (goat anti-mouse; Life Technologies), FITC (donkey anti-mouse; Millipore), Cy3 (donkey anti-rabbit; Millipore) and AlexaFluor594 (donkey anti-mouse IgM; Jackson Laboratories, USA). Following incubation, cells were washed twice more in 1X PBS and mounted with Fluoroshield anti-fade mounting media containing DAPI (Sapphire Bioscience, Australia). Negative controls included secondary antibody only (Chromeo488, Chromeo546, FITC, Cy3, AlexaFluor594) and isotype controls (donkey anti-mouse IgG, donkey anti-mouse IgM, donkey anti-rabbit IgG; Millipore). Primary and secondary antibodies and concentrations can be found in Table 1.

**Table 1:** Primary and secondary antibody concentrations for; Immunocytochemistry (ICC) and Fluorescence Assisted Cell Sorting (FACS).

Primary Antibody	Dilution	Application	Secondary Antibody	Dilution
**CD29**	1/100	ICC, FACS	Chromeo488	1/1000
**CD44**	1/500	ICC, FACS	Chromeo546	1/1000
**CD45**	1/500	ICC, FACS	Chromeo488	1/1000
**CD90**	1/200	ICC	Chromeo546	1/1000
**CD105**	1/200	ICC	Chromeo546	1/1000
**Nestin**	1/200	ICC, FACS	FITC	1/1000
**Sox2**	1/1000	ICC, FACS,	Cy3	1/1000
**GFAP**	1/500	ICC	Cy3	1/1000
**MAP2**	1/200	ICC	FITC	1/1000
**O1**	1/500	ICC	AlexaFluor594	1/500
**Mouse IgG**	1/1000	ICC	FITC/Chromeo546	1/1000
**Mouse IgM**	1/500	ICC	AlexaFluor594	1/500
**Rabbit IgG**	1/1000	ICC	Cy3/Chromeo488	1/1000

### RNA Isolation and Reverse Transcription

RNA was isolated from cells cultured in 6 well plates. Following culture, cells were washed twice in 1X PBS and placed on ice. To each well, 500μL of TRIzol (Invitrogen, Australia) was added to the cell monolayer, the monolayer homogenised and samples stored at -80°C for at least 24 hours. RNA was isolated using Zymo RNeasy MiniKit (Zymo, Australia) for RNA cleanup. Quality and quantity of RNA was assessed using Nanodrop (Thermo Fisher Scientific, Australia). For conversion to cDNA 100-150ng RNA was reverse transcribed with 10U Reverse Transcriptase (Roche, Australia), 1X RT buffer, 3μg random primers (Invitrogen, Australia), 1mM dNTPs (NEB, Australia) and 20U RNaseOUT (Invitrogen) in a 30μL reaction. Reverse transcription reactions were conducted in duplicate. Resulting cDNA was diluted to a working concentration of 40ng/μL.

### Q-PCR

We examined the gene expression of a number of markers commonly used to define MSCs and neural stem cells (NSCs). These included markers for each of the three mesenchymal lineages (bone, cartilage and fat) as well as markers specific to NSCs and early and late neural development. For the genes of interest, mRNA levels were quantified using Q-PCR (Life Technologies QuantStudio 7). Briefly, 120ng cDNA was amplified with 200μM primers (forward and reverse) with SYBR-Green PCR Master Mix (Promega, Australia) in a 10μL reaction. Cycling conditions were as follows: 50°C for 2 min, 95°C for 3 min then 50 cycles of 95°C for 3s, 60°C for 30s. Gene expression was normalised against expression of *18S* and calculated using 2^-ΔΔCt^. Specific primer sequences (IDT, USA) for the genes investigated can be found in S1 Table. Mean gene expression (2^-ΔΔCt^) was calculated between three independent experiments and graphed with SEM. All experiments were performed in quadruplicate.

#### Statistical Analysis of Q-PCR Data

All Q-PCR experiments on undifferentiated cells were performed in quadruplicate on biological triplicates. Experiments on differentiated neurospheres were conducted in triplicate. Variation between undifferentiated hMSCs and neurospheres was assessed using a two-tailed unpaired Student’s T-test assuming unequal variances. Mean gene expression (2^-ΔΔCt^) was calculated for each population and graphed with standard error of the mean (SEM). Statistical analysis was deemed significant when *P*<0.05.

### Fluorescence Activated Cell Sorting (FACS)

Cells were harvested using standard techniques. Briefly, growth media was removed from plates and cells washed once with 1X PBS followed by the addition of 5mL of trypsin (Gibco, Life Technologies) to the cell monolayer in a 100mm cell culture dish. Cells were incubated at 37°C for approximately 5 min, or until all cells had lifted from the plastic surface. To this, an equal volume (5mL) of trypsin neutralising media (TNS, culture medium + 10% foetal bovine serum) was added and the cells encouraged into a single cell suspension. Cells were counted using an automated cell counter (BioRad, Australia) and centrifuged at 650rcf for 5 min. Following this, trypsin/TNS was removed and the cell pellet resuspended in 5mL 1X PBS. Cells were centrifuged once more and the pellet resuspended in 4mL cold 1X PBS. To this, 6mL of ice cold 100% ethanol was added dropwise to the cells under gentle mixing and the cell suspension stored at 4°C for at least 30 min. Cells were then incubated in 1% BSA in 1X PBS for 15 min followed by blocking in 4% goat serum (GS) in 1X PBS for 15 min, centrifuged at 900rcf for 5 min and the pellet resuspended in 1% GS and incubated with primary antibody for 30 min at room temperature. All subsequent centrifugation steps were conducted at 900rcf for 5 min. Cells were washed in 1% BSA and incubated for 15min in 4% GS. Secondary antibodies (FITC/Cy3) were diluted in 1% GS and the cells incubated for 20 min at room temperature. Finally, cells were washed in 4% GS containing 0.1% TritonX-100 followed by a wash in 1% GS containing 0.3% Tween-20. The cells were resuspended in 1% GS and analysed on a BD FACS ARIA (BD Australia). Secondary antibody only negative controls were used for normalisation.

### Western Blotting

Total protein was extracted using protein-lysis buffer (20 mM HEPES, 25% Glycerol, 1.5 mM MgCl_2_, 420 mM NaCl, 0.5 mM DTT, 0.2 mM EDTA, 0.5% Igepal CA-630, 0.2 mM, 0.2 mM Na_3_VO_4_, 1 mM PMSF and Milli-Q-H_2_O containing protease and phosphatase inhibitors) and protein concentration determined using the BCA protein quantitation assay (Pierce). Approximately 50 μg of protein was separated by SDS-PAGE using 12% pre-cast gels (Mini-PROTEANTGX, Biorad), after which protein was transferred to a PVDF membrane using the Bio-Rad Transblot turbo system. The membrane was blocked with 5% milk, after which primary antibodies diluted in 5% BSA were added to the membrane and incubated on over night at 4°C. Primary antibodies used were: anti-CD29 (ab52971, Abcam, 1/500), anti-PPARγ (sc-7273, Santa Cruz Biotechnology, 1/200), anti-COL1A1 (sc-28657, Santa Cruz Biotechnology, 1/200), anti-TUBB3 (ab18207, Abcam, 1/1000) and anti-SOX2 (#AB5603, Millipore, 1/500) with anti-GAPDH (#2118, Cell Signaling, 1/1000) and anti-beta tubulin (#2128S, Cell Signaling, 1/1000) used as a loading control. The following day primary antibodies were removed and the membrane washed with PBST (1x PBS + 0.1% Tween-20) HRP-conjugated secondary antibodies (anti-Rabbit IgG, #7074 and anti-Mouse IgG, #7076, both from Cell Signaling used at 1/3000 dilution) were diluted in 5% BSA and the membrane incubated for 2h at room temperature. Detection of target proteins was performed with ECL (Clarity ECL, Bio-Rad) using the Fusion FX Spectra chemiluminescence system (Vilber Lourmat, Fisher Biotec).

## Results

### hMSCs Expansion

**Fig 1:**
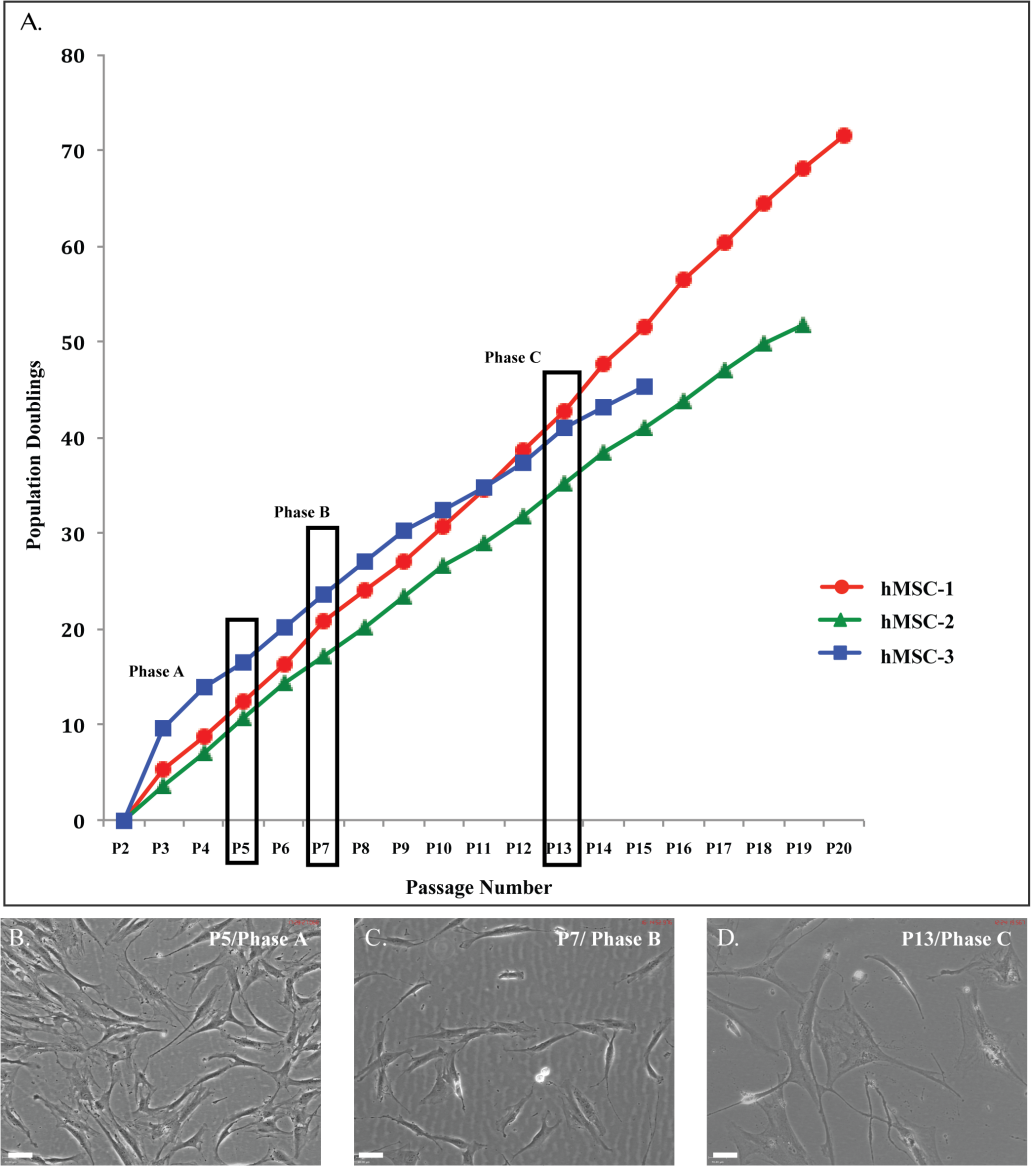
Expansion and morphology of hMSC populations. **A:** Growth curve of hMSC populations. Five different hMSC populations were expanded for more than 20 passages, 60 population doublings and 60 days in culture. Population doublings (PD) were calculated using the formula: PD = log [A/(BC)]/log2; where A was the number of collected cells, B was the number of plated cells and C was the attachment efficiency [41]. The five populations examined displayed similar patterns of growth allowing selection of three populations for ongoing experiments. Viability remained greater than 70% for all populations over this time. P+: passage; coloured lines represent each of three different hMSC populations selected for continuing experiments; red: hMSC-19604 (hMSC-1), green: hMSC-20176 (hMSC-2), blue: hMSC-21558 (hMSC-3). Representative phase contrast images of hMSC populations at **B:** P+5 **C:** P+7 (D) P+13 chosen to represent each growth phase (Phase A-C). Images shown at 10X magnification. Scale bar represents 70μm.

We successfully expanded commercially available hMSC populations from five different donors for more than 20 passages and 60 population doublings in maintenance media culture conditions. During expansion, cells underwent morphological changes and the cultures were monitored for growth and viability, with all populations demonstrating a multiphasic pattern of growth (Fig 1A). At P+5, cells had completed approximately 10 population doublings, while at P+7, this increased to 20 population doublings. Continued monitoring of expansion from P+7 to P+13, demonstrated a slower proliferation rate, with the cells completing a further 10 population doublings (approximately 30 by P+13). The proliferation rate was significantly slower after this passage with population doublings increasing to approximately 35 by P+15 with cultures maintaining viability >70% at all stages. At P+15, the proliferation rate slowed considerably with two of the populations ceasing to proliferate with no increase in cell number observed for more than 3 weeks. With all hMSC cultures demonstrating very similar morphological and growth characteristics we selected representative passages for each phase of growth from three of the five hMSC populations for more detailed analyses: Phase A—passage 5 (P+5) cells; Phase B—passage 7 (P+7) cells; and Phase C—passage 13 (P+13) cells. The donor populations identified for further examination are summarised in Table 2.

**Table 2:** Total days in culture and population doublings (PD) for individual hMSC populations. Total number of days in culture and population doublings calculated for each population at each passage representing each phase of growth. Average days in culture and population doublings for the three hMSC populations examined are presented.

Population	Age of Donor	Sex of Donor	Days in Culture P+5	PD P+5	Days in Culture P+7	PD P+7	Days in Culture P+13	PD P+13
**hMSC-19604 (hMSC-1)**	20	F	16	12	24	21	46	43
**hMSC-20176 (hMSC-2)**	33	M	9	11	16	17	35	35
**hMSC-21558 (hMSC-3)**	39	M	10	16	16	23	46	41
**Average**	**31**		**12**	**13**	**19**	**20**	**42**	**40**

Under maintenance culture conditions during Phase A (Fig 1B) the cultures were comprised of small, homogeneous fibroblastic-like cells with a rapid proliferation rate (3–4 days between passages). During Phase B (Fig 1C), the cells maintained their homogenous fibroblastic appearance with an increase in cell size and a slowing down of their proliferation rate (4–6 days between passages). By Phase C (Fig 1D), the cells became larger in size, with a reduced proliferation rate (7–14 days between passages), while maintaining their fibroblastic appearance with the cells becoming more heterogeneous. The cells were observed to enter into a stage of stasis with the cessation of proliferation accompanied by a flattening morphological change. However, one population (hMSC-1) demonstrated significant morphological changes with the appearance of colonies of small, rapidly proliferating cells of a more epithelial appearance (Fig 1A–P+16, P+19), with the cells appearing to escape terminal growth arrest. We have previously observed this in primary mammary epithelial cultures [41], however this, emergence from crisis only occurred in one of the hMSC populations examined. During the end of Phase C and entering Phase D (not shown) these smaller epithelial-like cells outnumbered the larger fibroblastic-like cells and the proliferation rate of the population increased (Fig 1A–P+19). While there was some observed variation in the total number of days in culture and population doublings between the populations, these differences were minor.

### hMSCs are positive for MSC markers throughout expansion, including CD45

**Fig 2:**
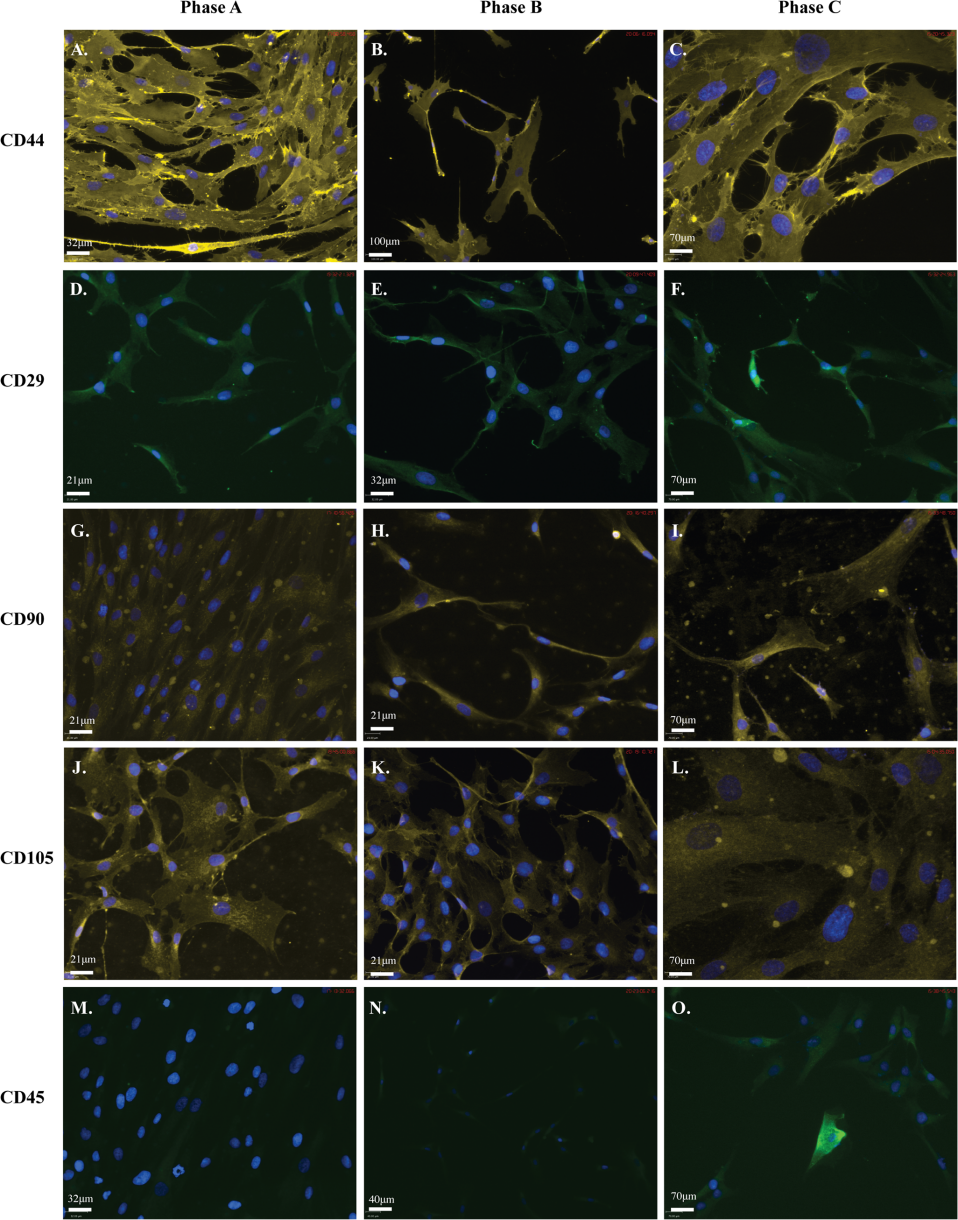
hMSC populations are positive for markers of MSC stemness. ICC showing positive staining for each of the five markers used to confirm hMSC stemness. Each of three hMSC populations was stained for each marker at each phase of growth. Cells are positive for: **A-C:** goat anti-mouse-CD44 (1/1000); **D-F:** goat anti-rabbit-CD29 (1/200); **G-I:** goat anti-mouse-CD90 (1/500); **J-L:** goat anti-mouse-CD105 (1/500); and negative for **M-O:** goat anti-rabbit-CD45 (1/500) at Phase A, Phase B and Phase C. At Phase C populations also become positive for CD45. Secondary antibodies used were anti-mouse-Chromeo546 (yellow; 1/1000) and anti-rabbit-Chromeo488 (green; 1/1000). Cells were mounted in mounting media containing DAPI (blue) to counterstain nuclei. Scale bar represents 70μm.

We next examined the cultures for MSC cell surface markers using a commercial panel of antibodies for detection by immunocytochemistry (ICC) and FACS. The panel consisted of antibodies against human CD44 (Fig 2A–2C), CD29 (alpha integrin, beta 1; Fig 2D–2F), CD90 (Thy-1; Fig 2G–2I), CD105 (Fig 2J–2L) and CD45 (Fig 2M–2O). ICC revealed specific staining for each of the positive markers of MSC stemness (CD105, CD90, CD44, CD29) while expression of the negative marker (CD45) was undetectable in Phase A cells (P+5) but increased to detectable levels in Phase C cells (P+13) (Fig 2; Table 3). Minimal background fluorescence was detected in isotype controls (ICC) and secondary antibody only controls (FACS) consistent with negative detection (not shown).

**Table 3:** Mesenchymal markers examined by FACS. Percentage of the cells positive for each MSC marker examined in MSCS at Phases A-C. Average percentage was calculated between the hMSC populations examined.

Marker	Population	Phase A	Phase B	Phase C
**CD29**	hMSC-20176	98%	98%	96%
	hMSC-21558	99%	98%	69%
	**Average**	**98.5%**	**98%**	**83%**
**CD44**	hMSC-20176	99%	99%	95%
	hMSC-21558	99%	98%	75%
	**Average**	**99%**	**98.5%**	**85%**
**CD45**	hMSC-20176	91%	93%	88%
	hMSC-21558	98%	97%	64%
	**Average**	**95%**	**95%**	**76%**

**Fig 3:**
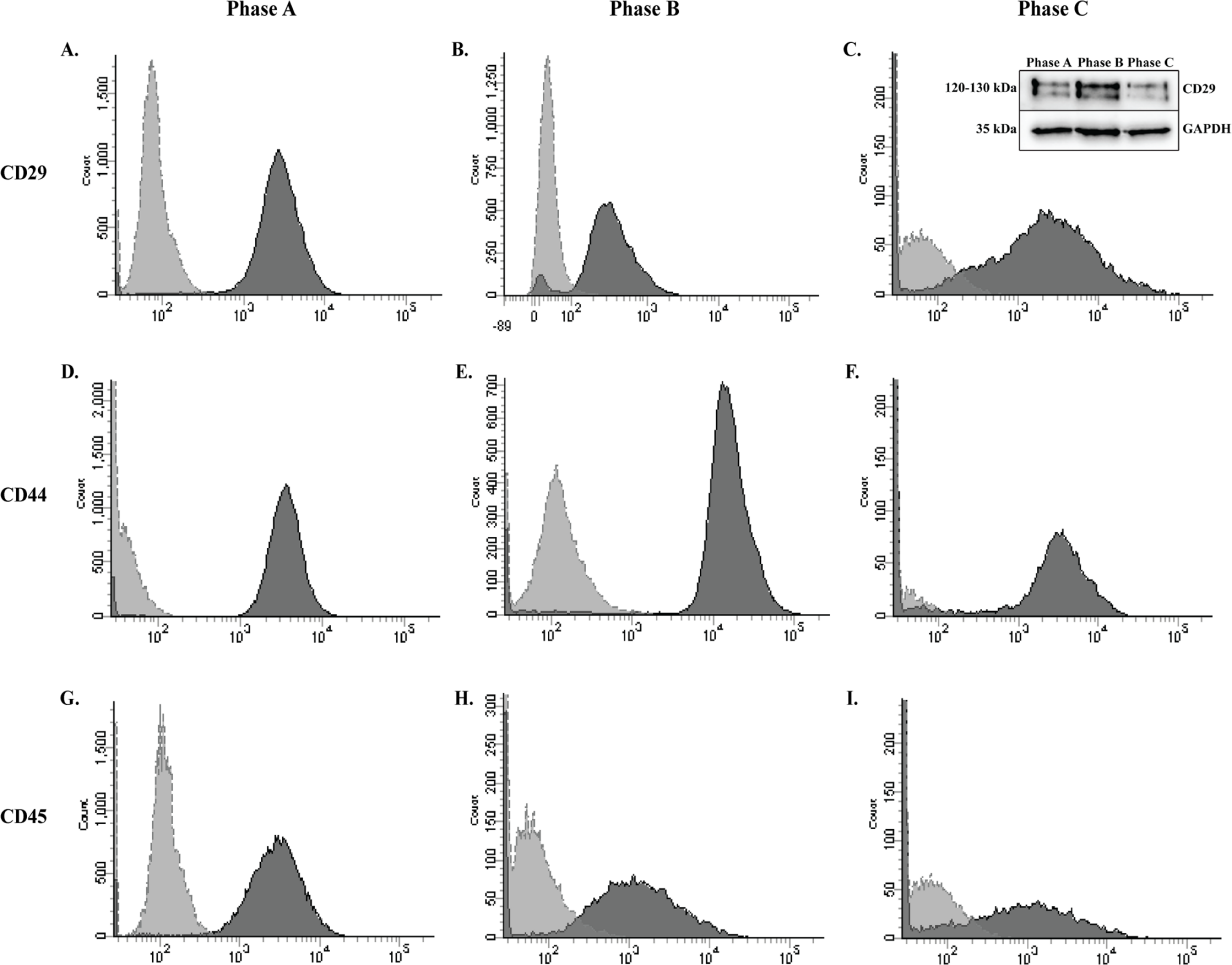
Mesenchymal lineage markers expressed in hMSC by FACS and Western Blotting. Detection by FACS demonstrates positive expression of three hMSC markers. Populations are more than 95% positive for **A-C:** CD29 and **D-F:** CD44. **G-I:** In addition, hMSC populations are more than 80% positive for the negative marker CD45 at each of the three passages examined during expansion. Dark grey histogram is the marker of interest. Light grey histogram is the secondary antibody only control. **Insert C:** The MSC marker CD29 (120-130kDa is present in hMSCs during all growth phases when examined by WB. GAPDH was used as a loading control.

We then examined the cells via FACS for a sub-selection of the MSC marker panel at growth phases A-C (Fig 3). FACS analysis determined the populations were >95% positive for established MSC markers (CD29 (A-C); CD44 (D-F)). Interestingly, >90% of the cells were also positive for the marker, CD45 (G-I). While among the most commonly cited negative markers of MSCs, cultures with low levels of CD45 have previously been reported [22,42]. Secondary antibody only staining of these cells produced negligible levels of auto-fluorescence against which samples were normalised. In addition Western blotting (WB) analysis confirmed CD29 protein in undifferentiated hMSCs at each growth phase (Fig 3 Insert C).

To ensure the cell surface maker profile was representative of commercially isolated and expanded hMSCs, expression of these markers was then validated by colleagues in additional (n = 2) hMSC populations from the same supplier by Q-PCR. Gene expression analysis supported our observations, with strong gene expression of CD44 and CD29 and low CD45 gene expression (10,000X lower than CD44) detected at all growth phases, with strong gene expression of CD105 and CD90 also observed (equal to CD44; data not shown). This data examining hMSC populations by ICC, FACS and Q-PCR suggest MSCs maintain their multipotentiality during expansion under standard culture conditions, but may demonstrate reduced or restricted lineage potential or efficiency of differentiation due to the continued expression of CD45.

### hMSCs retain mesenchymal lineage differentiative ability (Osteoblasts and Adipocytes) throughout expansion

**Fig 4:**
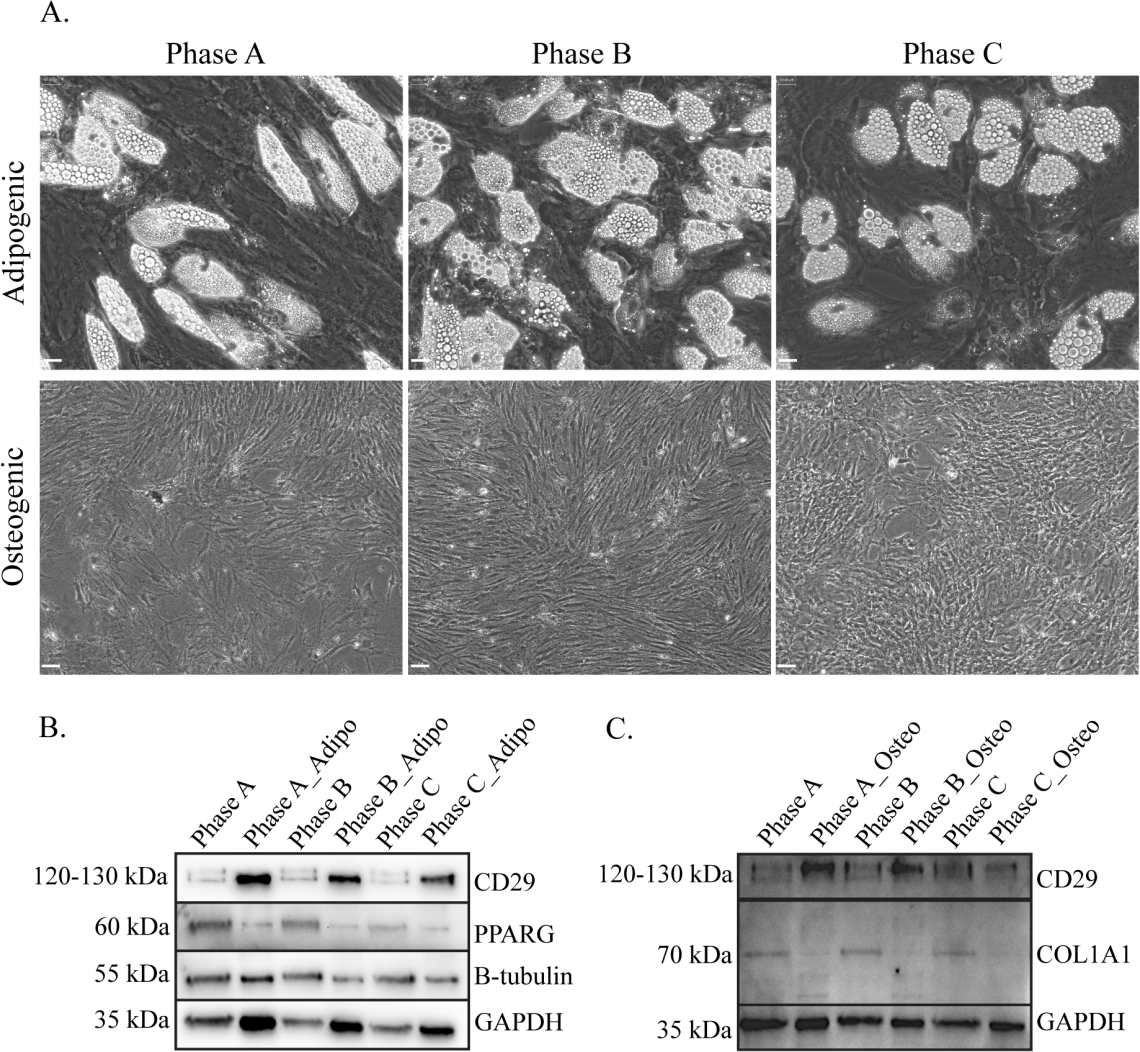
Adipogenic and osteogenic differentiation of MSCs at each passage during expansion. **A:** Phase contrast images of differentiated adipogenic and osteogenic cultures showing phenotypic changes as well as lipid droplet accumulation and mineralisation of the cultures. **WB analysis demonstrating protein detection of MSC markers CD29, PPARG and COL1A1 in undifferentiated hMSC at each growth phase** in **B:** differentiated Adipogenic cultures and **C:** Osteogenic cultures. GAPDH was used as the loading control. Scale bar represents 60μm.

Having established the hMSC cultures retain lineage potential and the associated cell surface marker profile for these cells, we next examined if the MSCs maintained multilineage potential during expansion. All hMSC populations included in the study were successfully differentiated toward adipogenic and osteogenic lineages at each growth phase. Adipocytes stained positive for Oil Red O (adipogenic stain) indicating the presence of lipid vacuoles, and osteoblast differentiation was confirmed by staining with Alizarin Red indicating mineralisation via the deposition of calcium (not shown). Although cultures were successfully differentiated at all phases, some variability was observed, with lower numbers of lipid vacuoles visible at Phase C (P+13) (Fig 4A) and reduced adipogenic staining (Oil Red O), however, hMSC cultures retain multilineage potential throughout expansion and into late growth phases. MSC osteoblast differentiation cultures demonstrated mineralisation (Fig 4A), confirmed via von Kossa staining (not shown). In addition, several MSC lineage proteins, collagen I (COL1A1; osteoblast) and peroxisome proliferator-activated receptor gamma (PPARG; adipocyte), were also detected by WB analysis in all phases of growth (Fig 4B and 4C). The mesenchymal marker CD29 was upregulated in both adipogenic and osteogenic cultures at all passages, with PPARG downregulated in adipogenic differentiated MSCs. Tubulin remained relatively stable following differentiation and collagen I (COL1A1) demonstrated decreased expression in osteogenic cultures with multiple bands observed in the differentiated cultures (Fig 4C).

### MSC marker gene expression throughout expansion

**Fig 5:**
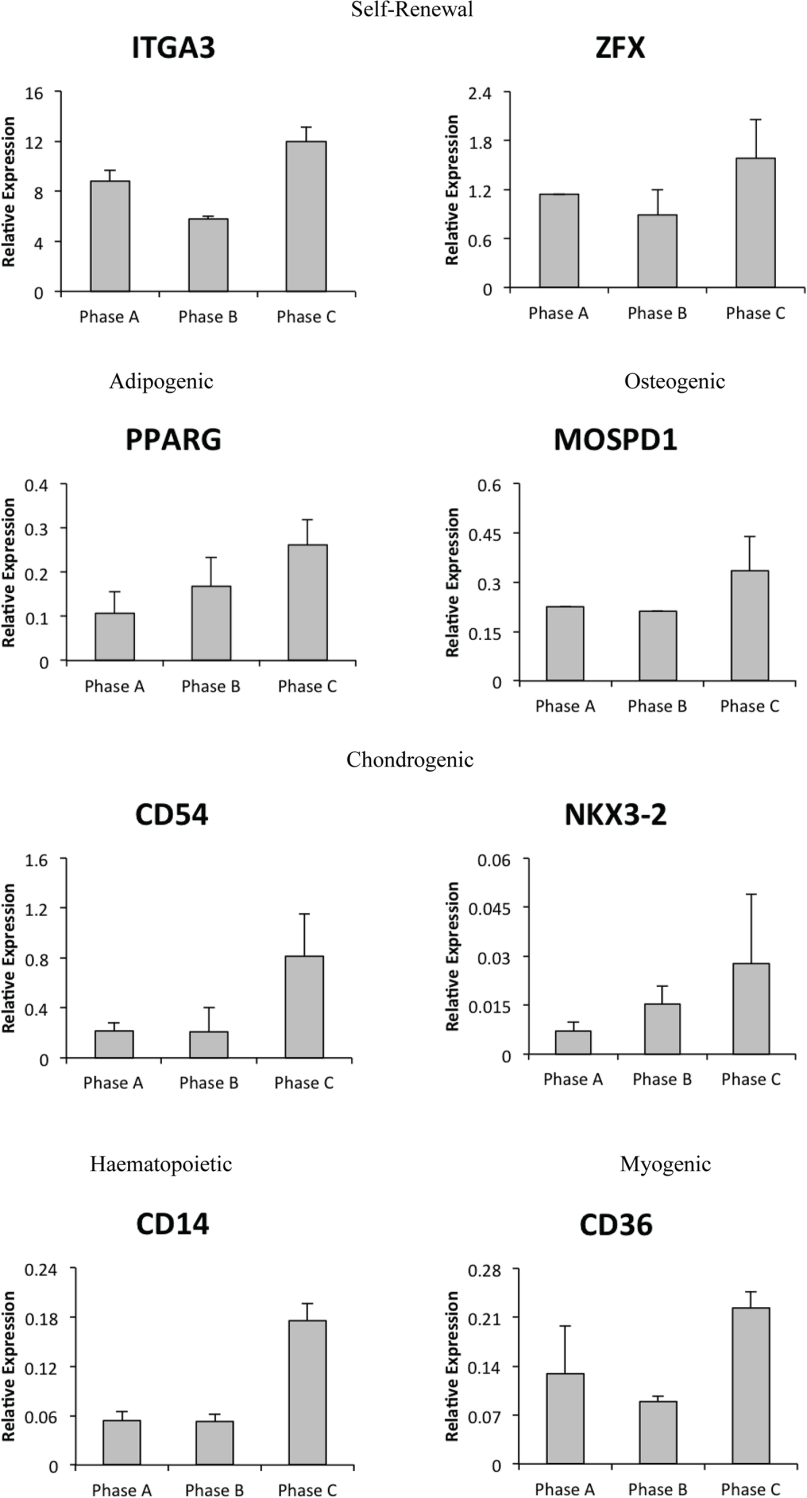
Gene expression changes of MSC cell surface markers and transcription factors. Several specific cell surface markers (CD14, CD26, CD54, ITGA3) and transcription factors (ZFX, PPARG, MOSPD1, NKX3-2) examined in extended hMSCs demonstrate increased expression in late growth phases.

Following confirmation cultures maintained stemness, adipogenic and osteogenic potential, to more fully define MSCs, we examined an extended panel of biomarkers (n = 56), transcription factors (n = 83) and cell surface markers (n = 69) by Q-PCR in the additional MSC cells. Results revealed that a number of cell surface markers (IGTA3, CD54, CD36 and CD14) and transcription factors (ZFX, PPARG, MOSPD1 and NKX3-2) traditionally used to identify MSCs increased expression during expansion (Fig 5).

**Fig 6:**
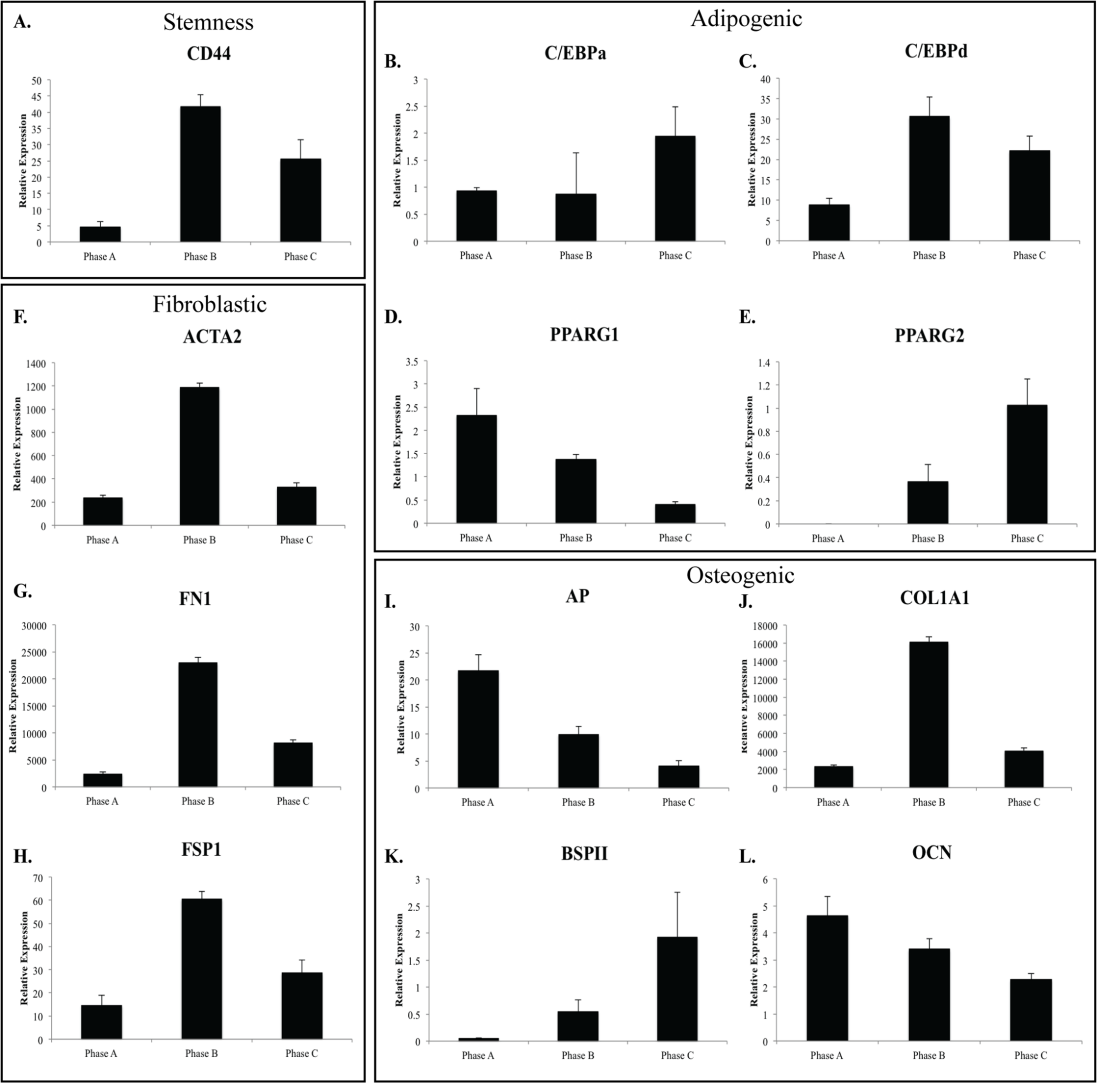
Relative gene expression of MSC lineage markers in hMSC populations throughout growth phases. Relative gene expression changes for **A:** Stemness markers (CD44); lineage specific **B-E:** adipogenic (C/EBPa, C/EBPd, PPARG1/2); **F-H:** Fibroblastic (ACTA2, FN1, FSP1); and **I-L:** osteogenic (AP, COL1A1, BSPII, OCN) in hMSCs (n = 3) at each passage. The majority of genes examined (lineage and stemness) increased expression at Phase B followed by decreased expression by Phase C (ACTA2, CD44, C/EBPd, COL1A1, FN1, FSP1). In contrast, lineage specific markers osteocalcin; OCN, Alkaline Phosphatase; AP and PPARG1 demonstrated decreased expression throughout expansion with bone sialoprotein II; BSPII, C/EBPa and PPARG2 demonstrated increased expression throughout expansion.

Further Q-PCR analysis revealed that hMSC populations continue to express MSC lineage specific genes at each phase of growth. Expression of bone specific markers alkaline phosphatase (AP) and osteocalcin (OCN) decreased throughout expansion. The adipogenic transcription factor peroxisome proliferator-activated receptor gamma (PPARG) was examined for each isoform, with isoform 1 (PPARG1) demonstrating decreased expression throughout expansion. In contrast, expression of bone sialoprotein II (BSPII) and PPARG isoform 2 (PPARG2) increased throughout expansion. Of the remaining adipogenic transcription factors examined, expression of C/EBPa remained relatively constant, while expression of C/EBPd peaked at Phase B (Fig 6). Notably expression levels of PPARG 1 & 2 was very low, approximately 10 times less than C/EBPd and observed expression of AP was much higher than both OCN and BSPII.

In addition to common markers of mesenchymal lineages, we also examined the expression of a number of other mesenchymal related genes. These included ACTA2, ADIPOQ, FSP1 (S100A4), FN1, COL1A1 and CDH2 (NCAD). With the exception of ADIPOQ, expression of these genes peaked in Phase B, following the expression pattern of C/EBPd. Smooth muscle actin (ACTA2), fibronectin (FN1) and the osteoblast lineage marker collagen 1 alpha 1 (COL1A1) were expressed at higher levels than the adipogenic transcription factors (C/EBP and PPARG) and osteocyte specific markers, AP, BSPII and OCN. Expression levels of the adipocyte lineage marker ADIPOQ was lowest at Phase B, similar to levels observed for adipogenic transcription (C/EBPa, C/EBPd, PPARG1, PPARG2) and osteocyte markers (OCN, BSPII). Interestingly, the observed continued expression of CD44 described above, a marker commonly used as a marker of MSC stemness, was expressed at a similar level to those of FSP1, C/EBP and PPARG2 but at a much lower level than COL1A1, FN1 and ACTA2 (Fig 6).

### Expression of neural cell surface markers and transcription factors

**Fig 7:**
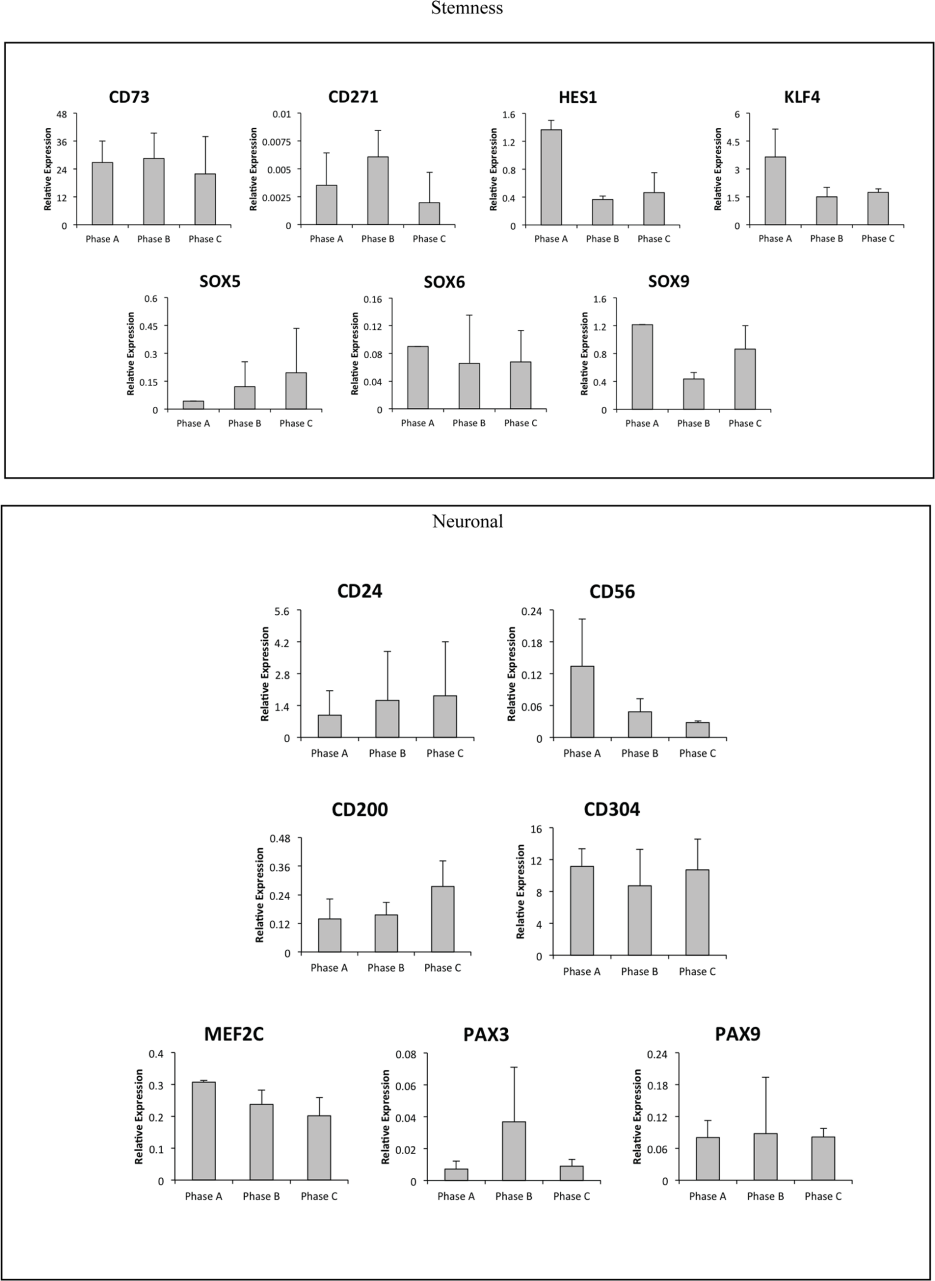
Neural transcription factors and cell surface CD marker expression. Undifferentiated hMSCs express transcription factors and cell surface CD markers including stemness (CD73, CD271, HES1, KLF4, SOX5/6/9) and neuronal (CD24, CD56, CD200, CD304, MEF2C, PAX3/9) markers reported to influence neural differentiation. With the exception of CD73 and CD304, expressed at levels equal to CD44 (a cell surface marker of MSCs), these neural markers were expressed at levels 10-10000X lower than CD44.

In addition to the mesenchymal markers examined in detail and described above, we investigated a number of transcription factors and cell surface (CD) markers reported to influence neural differentiation by Q-PCR. This data determined that hMSCs express a number of neural specific lineage markers including low expression levels for the neural transcription factors HES1, KLF4, MEF2C, PAX3 and PAX9 as well as SOX5, 6 & 9 in Phases A-C (Fig 7). In addition, cell surface markers used to aid in the identification of neural cells were also expressed at low levels including CD304, CD271, CD200, CD146, CD73, CD56 (NCAM) and CD24 (not shown).

### Neural markers throughout expansion

**Fig 8:**
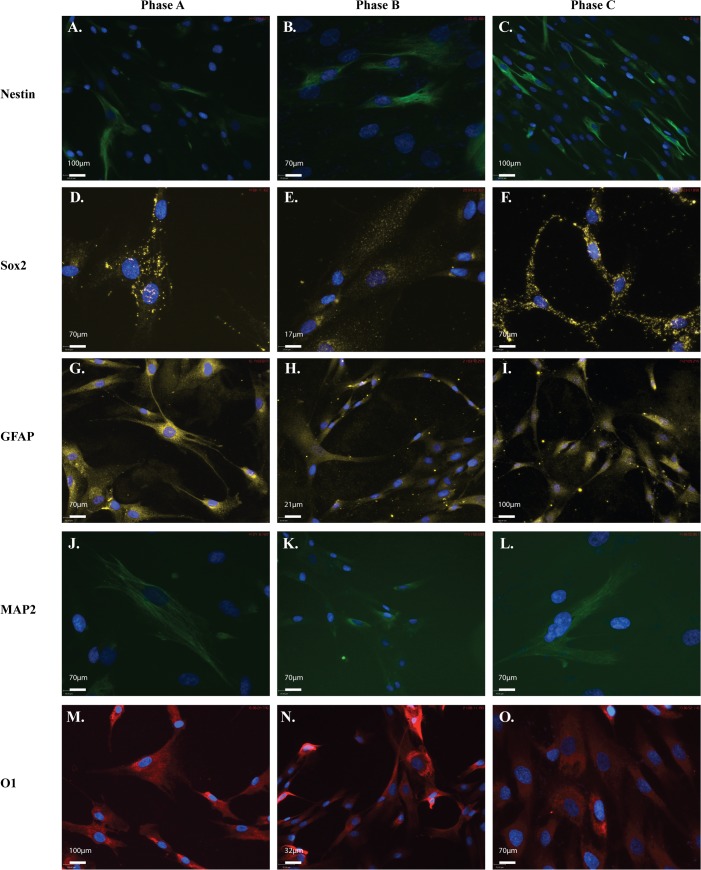
hMSCs are positive for neural markers throughout expansion. Representative ICC images showing positive neural marker staining for **A-F:** neural markers in each of the three MSC populations examined at each of the three passages investigated. Cells stained positive for: **A-C:** neural stem cell markers, donkey anti-mouse-Nestin (1/300); and **D-F:** donkey anti-rabbit-Sox2 (1/1000); as well as **G-I:** lineage markers donkey anti-rabbit-glial fibrillary acidic protein (astrocyte; GFAP; 1/500); **J-L:** donkey anti-mouse-microtubule associated protein 2 (neuronal; MAP2; 1/300); and **M-O:** donkey anti-mouse IgM-O1 (oligodendrocyte; 1/500). Secondary antibodies anti-mouse-FITC (green; 1/1000), anti-rabbit-Cy3 (yellow; 1/1000), anti-mouse IgM-AlexaFluor594 (red; 1/500). Cells were mounted in mounting media containing DAPI (blue) to counterstain nuclei. Scale bar represents 70μm.

We next examined the cultures for markers commonly used to identify neural stem cells (NSCs) and neural lineages by ICC. The maker panel consisted of Nestin, Sox2 (progenitor; Fig 8A–8C and 8D–8F), GFAP (astrocyte; Fig 8G–8I), MAP2 (neuronal; Fig 8J–8L) and O1 (oligodendrocyte; Fig 8M–8O) with hMSCs expressing these markers at each growth phase. Some variation in expression level between the populations was observed, with Sox2 expression seen to decrease at Phase B. Interestingly Sox2 localisation altered during expansion with a more nuclear expression observed at Phase B compared to the more cytoplasmic expression at both Phase A and Phase C. In contrast, most of the other makers remained at similar levels of expression at each passage. In order to quantify this, estimates were made by comparing the number of cells with positive staining to the total number of cells defined by DAPI stained nuclei. Nestin expression remained low with less than 50% of the population expressing this marker. The marker for mature neurons, MAP2 was expressed at a very low level with less than 2% of the population expressing MAP2 in ICC. In contrast, both the astrocyte marker GFAP, and the oligodendrocyte maker O1 were expressed in >80% of cells.

**Fig 9:**
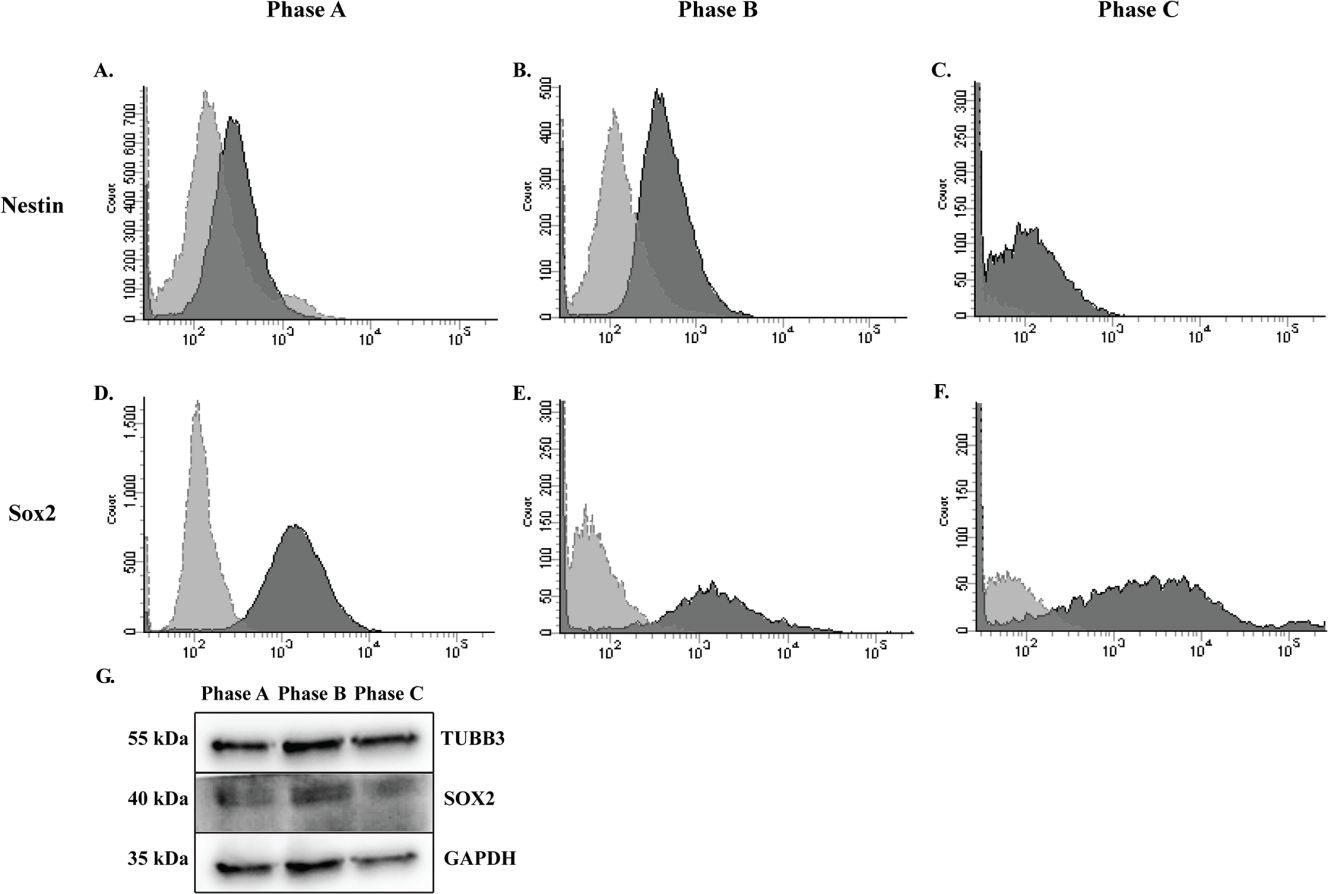
Neural marker proteins examined by FACS and WB in MSCs during expansion. Positive expression of neural markers is demonstrated. **A-C:** Populations average more than 30% positive for the neural stem cell marker, Nestin; and **D-F:** more than 50% positive for Sox2. Variation was observed between populations in percentages of the population positive for each maker, with the greatest variation seen at Phase B. Dark grey histogram is marker of interest. Light grey histogram is secondary antibody only control. **G:** Neural proteins TUBB3 and SOX2 were detected in undifferentiated hMSCs by WB during expansion.

We then confirmed ICC staining by FACS analysis of neural markers Nestin and Sox2 (Fig 9). The average percentage of cells positive for Nestin, as determined by FACS analysis increased throughout expansion (28%-48%), however, there was significant variation, particularly at P+7, between the two populations examined (Fig 9A–9C). Similarly, FACS analysis of Sox2 expression revealed a reduced percentage of the population positive at P+7 (49% on average) with increased expression at P+13 (90%) with substantial observed differences between the two populations at P+7 (Fig 9D–9F and Table 4). At each growth phase, we examined the neural proteins SOX2 and TUBB3 via WB analysis in undifferentiated hMSCs. Both markers remained relatively stable during expansion of MSCs with a decrease in Sox2 observed in Phase C cultures. Loading control was GAPDH (Fig 9G).

**Table 4:** Summary of NSC markers examined by FACS. Percentage of the population positive for each NSC maker. Average percentage was calculated between the hMSC populations examined.

Marker	Population	P+5	P+7	P+13
**Nestin**	hMSC-20176	42%	80%	61%
	hMSC-21558	14%	3%	34%
	**Average**	**28%**	**42%**	**48%**
**Sox2**	hMSC-20176	60%	2%	91%
	hMSC-21558	98%	95%	88%
	**Average**	**79%**	**49%**	**90%**

### hMSC neural gene expression profile

**Fig 10:**
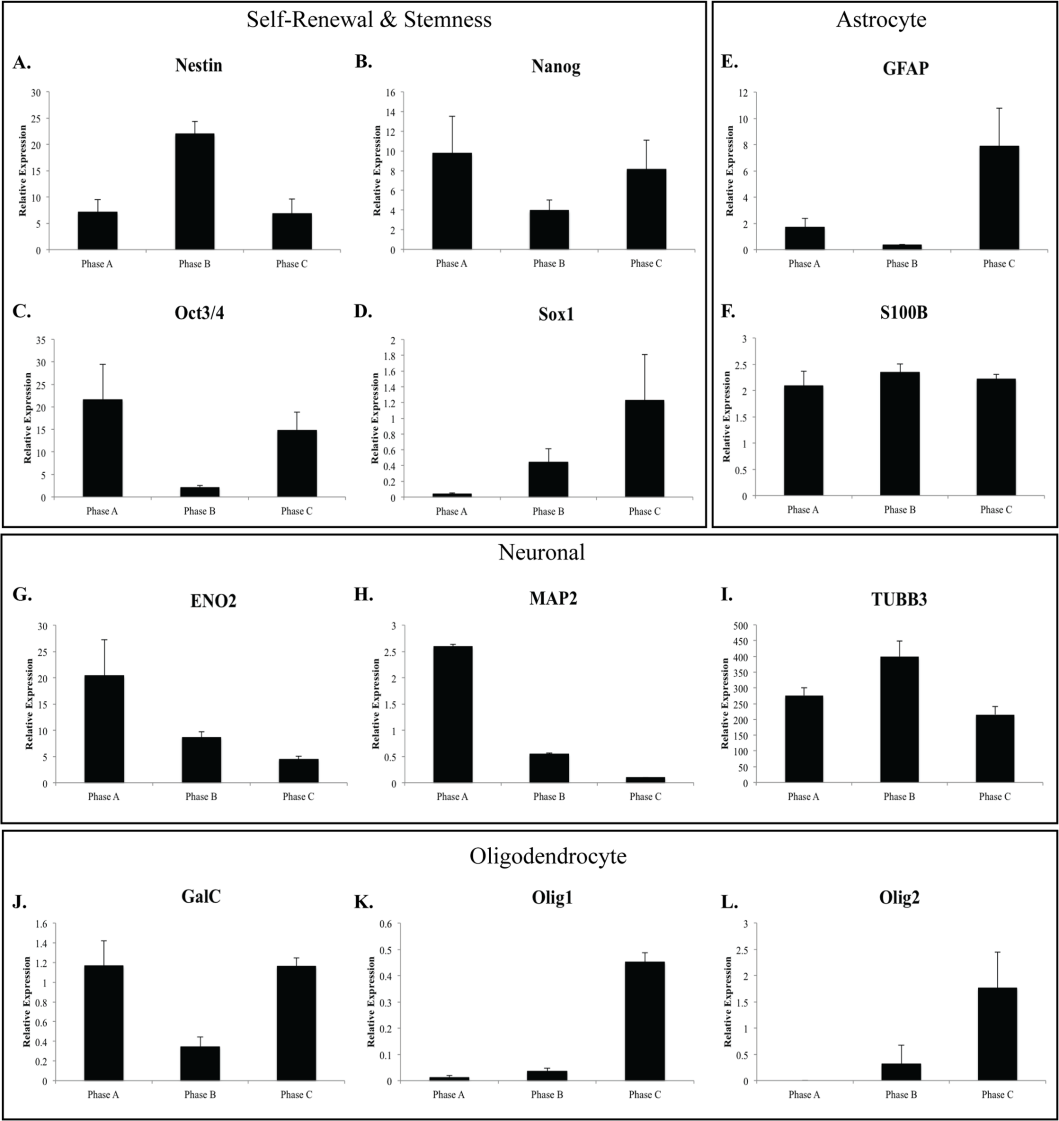
Relative gene expression of neural stem cell and lineage specific markers in hMSC populations throughout growth phases. **A-D:** Nestin, Nanog, OCT3/4 and SOX1 are markers of pluripotency in neural stem cells. Expression of these markers at each passage suggests the cells maintain pluripotency necessary for differentiating into different neural cell types. Expression of Nestin increased between Phase A and Phase B with a subsequent decrease in expression to Phase C. In contrast, both Nanog and OCT3/4 reduced expression at Phase B compared to Phase A and Phase C. Expression of each of these markers suggests the hMSC cultures maintain their ability to differentiate down each of the three neural lineages. Lineage specific markers examined include **E-F:** astrocyte markers GFAP and S100B; **G-I:** neuronal markers ENO2, MAP2 and TUBB3; **J-L:** Oligodendrocyte markers GalC, Olig1/2. Expression of MAP2 decreases over time, suggesting reduced ability to differentiate down a neural lineage. Expression of GFAP shows decreased expression at Phase B followed by a subsequent increase by Phase C. In contrast, TUBB3 expression increased between Phase A and Phase B cultures followed reduced expression at Phase C. These results may indicate optimal time points for differentiation to each of the neural lineages.

We then examined the gene expression of a number of neural markers in the hMSC cultures including markers used to define stemness in neural stem cells (Fig 10A–10D) as well as lineage specific markers (astrocyte, Fig 10E and 10F; neuronal, Fig 10G–10I; oligodendrocyte, Fig 10J–10L). Notably, there was variability in the expression level of the genes investigated between populations. The neural progenitor markers Nanog and Oct3/4 demonstrated similar expression patterns with a reduction in expression between Phase A and Phase B (0.2–0.5 fold) and a subsequent increase in expression at Phase C (3–4 fold). Interestingly, the astrocyte marker GFAP and the oligodendrocyte marker GalC followed a similar pattern, with expression of GFAP substantially higher in Phase C than Phase B cultures. The other astrocyte marker examined, S100B, showed a consistent expression level throughout expansion. Additional neural progenitor markers Nestin and Sox1 showed very different expression patterns. In contrast to the other neural genes examined, Nestin showed a peak in expression at Phase B, similar to the expression observed for NEFM (not shown) and β-III-tubulin. In contrast, Sox1 expression increased throughout expansion demonstrating a similar expression pattern to two oligodendrocyte markers, Olig1 and Olig2 (Fig 10). Both neuronal markers MAP2 and ENO2 were observed to reduce gene expression throughout expansion. Variability between populations was greatest in neural marker expression with Neurog2 (not shown), Sox1, Olig1 and Olig2, which were detected in only two of the three populations examined. No expression of DCX or MSI1 was detected.

### hMSC derived neurospheres

**Fig 11:**
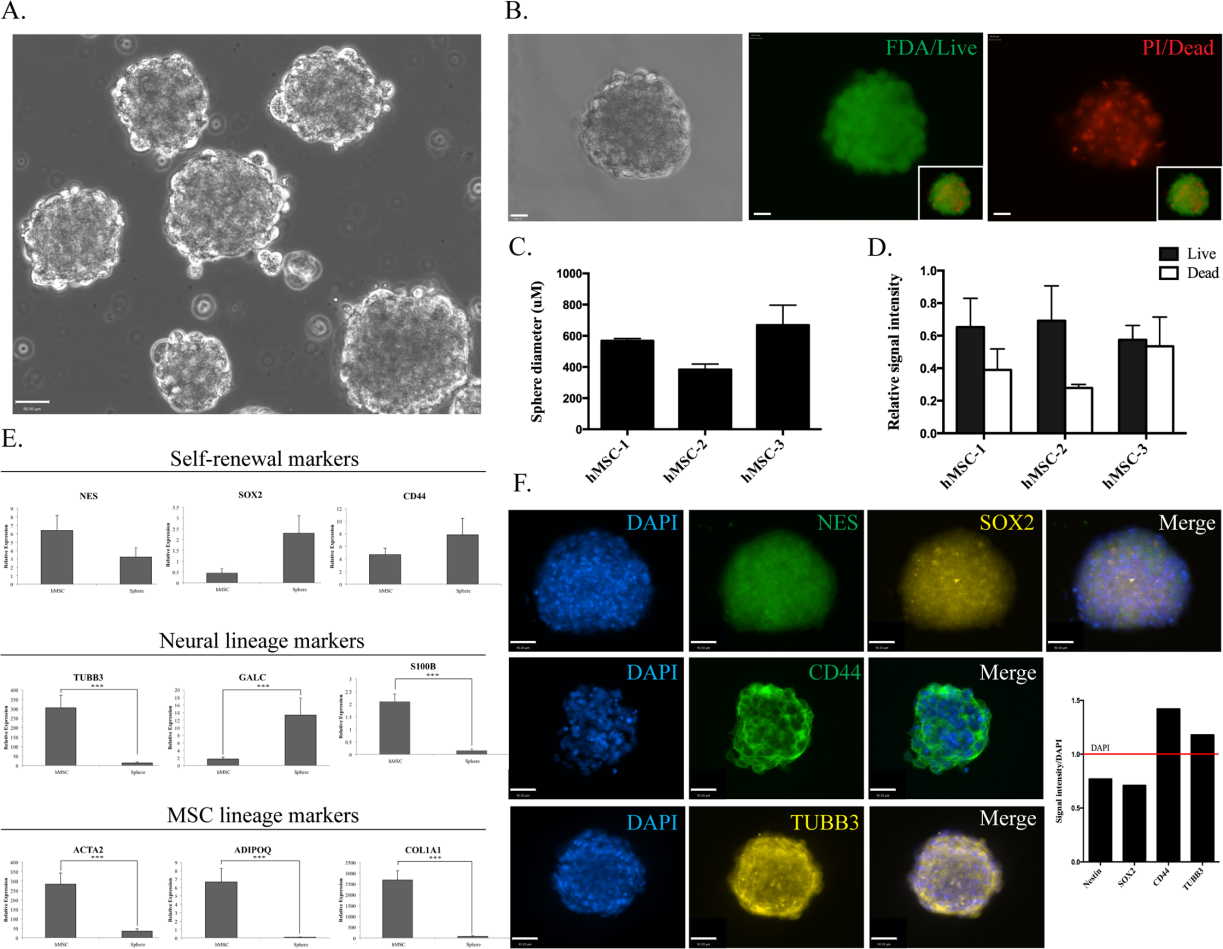
Characterisation of Phase A neurospheres. **A:** Phase contrast image (20x magnification, scale bar = 90 uM) of hMSC Phase A neurospheres **B:** FDA/PI stain indicating the presence of live (= green) and dead (= red) cells in hMSC neurospheres (40x magnification, scale bar = 90 uM). Bar graphs indicating **C:** the average sphere diameter for each hMSC population and **D:** the ratio of live/dead cells indicated by FDA and PI stain signal intensity. **E:** Q-PCR expression of self-renewal, neural and MSC lineage markers in undifferentiated Phase A hMSCs compared to neurospheres with significant differences in expression observed in multiple markers (*** p<0.001). F: ICC (40x magnification, scale bar = 90 uM) of Nestin, SOX2, CD44 and TUBB3 in neurospheres along with a bar graph indicating relative signal intensity of each of the markers when compared to DAPI.

In order to confirm neural lineage potential, hMSCs were differentiated into neurospheres at Phase A. Spheres were observed as little as one hour after plating into differentiation conditions. Over subsequent hours, sphere number and size steadily increased to a maximum size of around 600μm (Fig 11A–11C). The ratio of live to dead cells within the spheres was determined by FDA/PI staining. Live cells (positive FDA (green) staining, dead cells positive for PI (red); Fig 11B) for each population, demonstrated the spheres consisted of more than 50% live cells (Fig 11D).

We then examined the gene expression profile of the spheres including a number of MSC and neural makers previously examined in undifferentiated hMSCs (Fig 11E). Nestin, a marker of neural stemness demonstrated a non-significant reduction in gene expression (p = 0.192460416) in neurospheres compared to hMSCs while SOX2 demonstrated increased expression in neurospheres (p = 0.230056363). In contrast, the MSC marker of stemness, CD44, demonstrated a non-significant increase in expression in neurospheres when compared to undifferentiated hMSCs (p = 0.218534045). Of the neural lineage markers examined, the early neuronal marker TUBB3 demonstrated reduced expression in neurospheres (p = 0.00013324), as did the astrocyte maker, S100B (p = 1.20405E-06). In contrast, the oligodendrocyte maker, GALC, demonstrated increased expression in neurosphere cultures (p = 0.000431688). MSC lineage markers, ACTA2, ADIPOQ and CO1A1 demonstrated significantly decreased expression in neurospheres when compared to undifferentiated hMSCs (ACTA2: p = 0.000218789; ADIPOQ: p = 0.000327476; COL1A1: p = 2.16575E-06).

When examined by ICC, the MSC neurospheres clearly demonstrated positive staining for Nestin, Sox2, CD44 and TUBB3 (Fig 11F). ICC and FACS analysis of undifferentiated cells determined that on average, less than 50% of hMSCs were positive for Nestin, while ICC staining of spheres suggests all cells within the sphere are positive for this marker. The number of undifferentiated hMSCs staining positive for Sox2 was elevated (50–90%) and this high percentage of positive stained cells was maintained in neurospheres with more than 90% of cells positive.

## Discussion

Human MSCs co-express mesenchymal and neural markers throughout expansion, however, this does not inhibit their ability to differentiate toward adipogenic and osteogenic lineages or their ability to form neurospheres. This study contributes to the growing body of evidence including work by Wetzig *et al*, demonstrating common MSC markers do not discriminate between MSC and non-stem cell mesenchymal cultures [43]. In order to improve MSC therapeutic efficacy, there is a need for the identification of better MSC markers encompassing cell surface and gene expression to identify and control potency [20] and lineage potential.

While some cells examined in this study by ICC continued to express the haematopoietic marker, CD45, this expression was low in the majority of hMSC donor populations examined when compared with FACS staining of CD44 and CD29 and by gene expression analysis including the extended donor cultures. This correlates with the CD45^low^ expression reported previously by Yu *et al* where both CD44 and CD45 were reported in MSCs at low levels [42] supporting CD45^+^ direct selection of mesenchymal stem cells [44]. Depleting fresh bone marrow of haematopoietic cells (non-MSC) using negative selection based on CD45 [45] resulted in the fibroblastic colony forming units (CFU-F) detected in the CD45^+^ fraction [44]. Why unprocessed MSCs were shown to be CD45^+^ while cultured or more mature MSCs were CD45^-^ may in part be explained by different CD45 isoforms [46]. Expression of CD45 by haematopoietic stem cells (HSCs) and MSCs combined with their bone marrow localisation suggest that these cells are perhaps more closely related than previously reported [44]. Some evidence suggests that the co-existence of haematopoietic stem cells with MSCs provides cooperation for differentiation to both cell types [47,48] including an early study by Singer *et al*, where bone marrow stromal cell lines were generated that expressed haematopoietic markers [49]. Indeed, the consistently observed increase of CD45 in hMSCs during expansion did not diminish their osteogenic and adipogenic lineage-specific differentiation or alter expression of their associated lineage markers. However, there remains inconsistency regarding the accepted cell surface profile of hMSCs [22].

The commercial identification panel used in this study was comprised of five of the currently accepted most common markers used in the identification and characterisation of MSCs–CD105, CD90, CD44, CD29 (positive) and CD45 (negative). Several markers previously reported primarily for the identification of other types of stem cells have also been reported to be detected in MSC populations, including Nanog and Sox2, which are generally accepted as neural progenitor markers [22,50]. In addition to cell surface markers, several transcription factors have been reported in the identification of MSC and neural stem cells (NSCs) for understanding the lineage specific potential of stem cell progenitor cells as well as the identification of stem cell type. In MSCs, the transcription factor HES1 has been reported to inhibit adipogenesis [51] while in NSCs HES1 has been demonstrated to have roles in neurosphere proliferation and neuronal differentiation [52]. Cell surface markers such as β-1 integrin CD29 are used in the identification of MSCs and as a neural differentiation antigen on proliferating NSC-derived neurons [53]. Other cell surface markers used in the identification of both MSCs and NSCs include CD146, CD73, CD56 (NCAM) and CD24. Transcription factors reported in both cell types include HES1 (maintenance of stemness), KLF4 (growth & development [54]), MEF2C (neuronal morphogenesis [55]), as well as PAX family (lineage specific proliferation, migration and neural development [56,57]) and SOX family (stem cell maintenance [58]) genes.

Several studies have examined the neural potential of MSC demonstrating expression of neural markers in undifferentiated and unstimulated conditions [32,33]. However, most studies, with the exception of this study and our previous study [32] which reported expression of both mesenchymal and neural markers in commercially obtained MSC cultures, have used freshly isolated MSCs from bone marrow aspirates [33] or other sources, including adipose tissue and dental pulp [11]. Interestingly, a more specific neural marker expression in undifferentiated MSCs throughout expansion has recently emerged in murine cells. Foudah *et al* (2012) reported the presence of neural markers between P+0 (Phase A equivalent cultures) and P+40 (Phase C equivalent cultures) following isolation of rat MSCs from bone marrow [37] with a greater number of cells expressing Nestin towards the middle of the expansion period followed by a decline in positive cell numbers [37]. In addition they also reported a steady decline in GFAP positive cells throughout expansion [37]. A similar trend is observed in the expanded donor hMSCs with the peak in Nestin gene expression observed in the middle of the expansion (Phase B). In addition, both studies report similar results for β-III-tubulin, with the peak in gene expression similarly occurring in early to mid cultures, with the peak expression in the rat MSCs occurring earlier than the human cells, in Phase A equivalent cultures [37]. This demonstrates heterogeneity in the MSC cultures, with some cells expressing neural markers while others do not, suggesting subpopulations of cells with varying differentiation capacity. In addition, this data also highlights our current lack of knowledge in terms of the neural lineage capacity of hMSCs. In order to broaden the therapeutic potential of these cells, controlling lineage commitment and specification is needed to maximise their differentiation capacity.

However, the complexity of defining cells by their origin and function is not limited to MSCs. Neural lineage markers are also expressed in a wide range of cell types including murine MSCs, osteoblasts and adipocytes [33]. β-III-tubulin, an early neuronal marker, is expressed in embryos, adults [59,60], tumour cells [61,62] and as a component of the mitotic spindle [63]. Nestin, considered a neural progenitor marker [64] has also been found in extra-neuronal tissues [65] including human solid tumours [66] and has been used as a predictor of poor prognosis in malignant melanoma [67]. Markers of mesodermal-specific lineages such as the osteogenic markers osteopontin and osteocalcin along with the adipogenic marker PPARG have been detected, in turn, in neural cells [68,69]. In addition, one of the most commonly reported MSC positive cell surface markers, CD44, is also reported to be a marker of immature astrocytes [70–72]. Other studies have demonstrated astrocyte-restricted precursor cells isolated from the glia rich postnatal day 3 mouse cerebellum to have high CD44 expression [70], while CD44^+^ astrocyte-restricted precursor cells have been identified in the developing rodent spinal cord prior to the acquisition of positive GFAP immunoreactivity [72]. The involvement of CD44 in neural development is not limited to the astrocyte lineage, with an important role for CD44 in oligodendrocyte differentiation supported by decreased oligodendrocyte maturation in CNP-CD44 transgenic mice overexpressing CD44 [72,73].

This study has demonstrated that commercially isolated hMSCs have shorter doubling times (4–7 days between passages) than freshly isolated cultures (2 weeks between passages; [33]). It is possible that commercially isolated cells are a more homogeneous representative population, removing the confusion that heterogeneous freshly isolated populations bring to the definition and characterisation of hMSCs. In addition, although expression of neural markers is suggestive of neural potential in hMSCs it is not evidence of the cells’ ability to differentiate toward neural lineages. However, the formation of neurospheres along with associated neural lineage marker expression changes confirm the MSCs have and maintain neural potential during expansion. The observed morphological changes however do not demonstrate functionality, and this is an important focus of future work. While staining techniques examining ion uptake may indicate restricted neuronal function and morphological changes indicative of neural differentiation, physiological examination of differentiated cells is required to confirm functionality. To establish electro-physiological neuronal function, extended *in vitro* culture (likely > 100 days) is required. However, evidence provided here suggests that there are optimal *in vitro* intervals and some key marker expression interactions that may help to achieve this goal.

While the evidence suggests that due to its widespread and variable expression, CD44 is not a suitable marker to differentiate between MSCs and other cell types, it is important to remember that there are a number of isoforms of CD44 generated by alternative splicing of the extracellular domain [74]. This alternative splicing is tissue-specific [75] with most normal tissues expressing the standard form [74,76]. However, in haematopoietic cells, alternative splicing of exons has been induced by cell activation or treatment with inflammatory cytokines such as interleukin-1 (IL-1) [77–81]. Further, CD44 isoforms carrying heparan sulfate chains have been demonstrated to regulate fibroblast growth factor activation [74], an important signalling pathway in MSC differentiation. Determining the specific CD44 isoform present in neural cells compared to MSCs would refine its’ usefulness to differentiate between MSCs and other cell types.

## Conclusion

It is becoming increasingly clear that it is not only the cell surface profile of MSCs that is important for their definition and characterisation, but also the context (cellular niche) from which they have been isolated. In order to realise the full therapeutic potential of these cells we must distinguish between origins of MSCs–bone marrow-, adipose-, dental pulp-derived–to establish how this affects their targeted application. To achieve this, perhaps only in the short term, the use of commercially isolated hMSCs with their reduced variability in isolation may provide a homogenous cell population enabling detailed, consistent characterisation of these valuable cells. Our study and others indicate the need for standardised isolation, characterisation and definition of MSCs for the analyses of biomarkers and gene expression necessary for adequate definition of these cells. In addition, our study has demonstrated the continued lineage multipotentiality of MSCs during extended culture including neural lineages. Our ability to limit variability and improve therapeutic efficacy through better defined cultures will provide much needed improvements in the translation of MSCs to the clinic for increased reproducibility and routine production of MSCs for therapeutic applications.

## Supporting Information

S1 Table Primer Sequences. Primer sequences for Q-PCR conducted in this study.
(DOCX)
