# Supplementary material for: Human Mesenchymal Stem Cells Retain Multilineage Differentiation Capacity Including Neural Marker Expression after Extended In Vitro Expansion
Source: PLoS One. 2015 Sep 10;10(9):e0137255. doi: 10.1371/journal.pone.0137255 (PMC4565666; doi:10.1371/journal.pone.0137255)
Supplement: S1 Table — Primer sequences for Q-PCR conducted in this study. (DOCX) [file pone.0137255.s001.docx]

***Supplementary Table 1. Primer Sequences***

| Gene | Symbol | Forward Primer | Reverse Primer | Amplicon (bp) | Accession Number | Lineage |
| --- | --- | --- | --- | --- | --- | --- |
| Smooth Muscle Actin | ACTA2 | TAAGACGGGAATCCTGTGAAGC | TACAGAGCCCAGAGCCATTG | 90 | NM_001141945.1 | Myogenic |
| Adiponectin, C1Q and collagen domain containing | ADIPOQ | AAAGGAGATCCAGGTCTTATTGGT | GTTCTCCTTTCCTGCCTTGGA | 109 | NM_001177800.1 | Adipogenic |
| Fibronectin | FN1 | ATACCATCATCCCAGAGGTGC | TGGTGGAAGAGTTTAGCGGG | 102 | NM_212482.1 | Osteogenic |
| CD44 | CD44 | AGCAACTGAGACAGCAACCA | AGACGTACCAGCCATTTGTGT | 115 | NM_000610.3 | MSC |
| S100 Calcium binding protein A4 (FSP1) | S100A4 | CTTGGTTTGATCCTGACTGCTG | ACTTGTCACCCTCTTTGCCC | 103 | NM_002961.2 | Osteogenic |
| N-Cadherin (NCAD) | CDH2 | GAGGGATCAAAGCCTGGAACA | TTGAGGGCATTGGGATCGTC | 71 | NM_001792.3 | Neural |
| Collagen type 1, alpha 1 | COL1A1 | ACATGTTCAGCTTTGTGGACC | TGATTGGTGGGATGTCTTCGT | 117 | NM_000088.3 | Osteogenic |
| Nestin | NES | CTCAGCTTTCAGGACCCCAA | GTCTCAAGGGTAGCAGGCAA | 128 | NM_006617.1 | NSC |
| Nanog homeobox | NANOG | ACCTCAGCTACAAACAGGTGAA | AAAGGCTGGGGTAGGTAGGT | 135 | NM_024865.2 | Progenitor |
| POU Class 5 homeobox 1 (OCT3/4) | POU5F1 | ATCTTCAGGAGATATGCAAAGCAGA | TGATCTGCTGCAGTGTGGGT | 135 | NM_002701.4 | Progenitor |
| Microtubule Associated Protein 2 | MAP2 | GACTGCAGCTCTGCCTTTAG | AAGTAAATCTTCCTCCACTGTGAC | 106 | NM_002374.3 | Neural |
| Glial Fibrillary Acidic Protein | GFAP | GCGGGATGGAGAGGTCATTA | CAGAGGCGGAGCAACTATCC | 131 | NM_002055.4 | Astrocyte |
| β-III Tubulin | TUBB3 | GGCCAAGTTCTGGGAAGTCAT | CTCGAGGCACGTACTTGTGA | 137 | NM_006086.3 | Neural |
| Enolase 2 | ENO2 | TGCACAGGCCAGATCAAGAC | ACAGCACACTGGGATTACGG | 139 | NM_001975.2 | Neural |
| SRY (sex determining region Y) box 1 | SOX1 | CAACCAGGACCGGGTCAAAC | CCTCGGACATGACCTTCCAC | 146 | NM_005986.2 | Neural |
| Alkaline Phosphatase | AP | ATGCCCTGGAGCTTCAGAAG | TGGTGGAGCTGACCCTTGAG | 121 | NM_000478 | Osteogenic |
| Bone Sialoprotein II (ISBP) | BSPII | AGAGGAAGCAATCACCAAAATGA | TTGAGAAAGCACAGGCCATTC | 74 | NM_004967 | Osteogenic |
| CCAAT/enhancer binding protein alpha | C/EBPa | GAGGGACCGGAGTTATGACAAG | GGCACAGAGGCCAGATACAAG | 80 | NM_004364.2 | Adipogenic |
| CCAAT/enhancer binding protein delta | C/EBPd | CATAGGAGCGCAAAGAAGCTACA | TTAGCTGCATCAACAGGAGTAAGATG | 135 | NM_005195.2 | Adipogenic |
| Osteocalcin | OCN | GGTGCAGAGTCCAGCAAAGG | CGCCTGGGTCTCTTCACTACC | 74 | NM_199173 | Osteogenic |
| Peroxisome proliferator-activated receptor gamma 1 | PPARG1 | GGCCGCAGATTTGAAAGAAG | ATTTCGTTAAAGGCTGACTCTCGTT | 86 | NM_138712 | Adipogenic |
| Peroxisome proliferator-activated receptor gamma 2 | PPARG2 | TGTCTTGACTCATGGGTGTATTCA | ACACTGATATTTTGTCTCTATCTAGCAAAAG | 97 | NM_015869 | Adipogenic |
| S100 Calcium binding protein B | S100B | TTCTGGAAGGGAGGGAGACA | CTCCTGCTCTTTGATTTCCTCT | 103 | NM_006272.2 | Astrocyte |
| Galacto-sylceramidase | GalC | GCCAAGCGTTACCATGATTT | TTTCACTCGCTGGAGACCTT | 123 | NM_001201402.1 | Oligodendrocyte |
| Oligodendrocyte transcription factor 1 | Olig1 | GTCATCCTGCCCTACTCAGC | CTGCCCAGCAGTAGGATGTAG | 107 | NM_138983.2 | Oligodendrocyte |
| Oligodendrocyte transcription factor 2 | Olig2 | GACAAGCTAGGAGGCAGTGG | CGGCTCTGTCATTTGCTTCT | 111 | NM_005806.3 | Oligodendrocyte |
| Integrin, alpha 3 (CD49C) | ITGA3 | CTACCACAACGAGATGTGCAA | CCGAAGTACACAGTGTTCTGG | 102 | NM_002204.2 | Self-Renewal |
| Zinc finger protein, X-linked | ZFX | TTGCTGAAATCGCTGACGAAG | GCAATCGGCATGAAGGTTTTGAT | 133 | NM_003410.3 | Self-Renewal |
| Peroxisome proliferator-activated receptor gamma | PPARG | TGGAATTAGATGACAGCGACTTGG | CTGGAGCAGCTTGGCAAACA | 182 | NM_138712.3 | Adipogenic |
| Motile sperm domain containing 1 | MOSPD1 | TGTGATTCGTCATCGAGATGTTC | CTTTGCTCGGAAACTTGGAGA | 78 | NM_019556.2 | Osteogenic |
| Intercellular adhesion molecule 1 (ICAM1) | CD54 | ATGCCCAGACATCTGTGTCC | GGGGTCTCTATGCCCAACAA | 112 | NM_000201.2 | Chondrogenic |
| NK3 homeobox 2 | NKX3-2 | cgacgaccagagacaatacct | aatagtaggagggctgcagtg | 77 | NM_001189.3 | Chondrogenic |
| CD14 molecule | CD14 | CAACCTAGAGCCGTTTCTAAAGC | GCGCCTACCAGTAGCTGAG | 135 | NM_000591.3 | Haematopoeitc |
| CD36 molecule | CD36 | CTTTGGCTTAATGAGACTGGGAC | GCAACAAACATCACCACACCA | 134 | NM_001001548.2 | Myogenic |
| 5'-nucleotidase, ecto (NT5E, CD73) | CD73 | AAGGACTGATCGAGCCACTC | GGAAGTGTATCCAACGATTCCCA | 161 | NM_002526.3 | Stemness |
| Nerve growth factor receptor (NGFR, CD271) | CD271 | CCTACGGCTACTACCAGGATG | CACACGGTGTTCTGCTTGT | 109 | NM_002507.3 | Stemness |
| HES family bHLH transcription factor 1 | HES1 | CCTGTCATCCCCGTCTACAC | CACATGGAGTCCGCCGTAA | 98 | NM_005524.3 | Stemness |
| Kruppel-like factor 4 | KLF4 | AAGAGTTCCCATCTCAAGGCACA | GGGCGAATTTCCATCCACAG | 91 | NM_004235.4 | Stemness |
| SRY (sex determining region Y)-box 5 | SOX5 | CAGCCAGAGTTAGCACAATAGG | CTGTTGTTCCCGTCGGAGTT | 104 | NM_006940.4 | Stemness |
| SRY (sex determining region Y)-box 6 | SOX6 | GGATGCAATGACCCAGGATTT | TGAATGGTACTGACAAGTGTTGG | 141 | NM_017508.2 | Stemness |
| SRY (sex determining region Y)-box 9 | SOX9 | TGTATCACTGAGTCATTTGCAGTGT | AAGGTCTGTCAGTGGGCTGAT | 187 | NM_000346.3 | Stemness |
| CD24 molecule | CD24 | CTCCTACCCACGCAGATTTATTC | AGAGTGAGACCACGAAGAGAC | 166 | NM_013230.3 | Neuronal |
| Neural Cell AdhesionMolecule 1 (NCAM1, CD56) | CD56 | GGCATTTACAAGTGTGTGGTTAC | TTGGCGCATTCTTGAACATGA | 100 | NM_000615.6 | Neuronal |
| CD200 molecule | CD200 | ACGTCTGTTACCAGCATCCTC | CTTAAAGTCGGTCACAGTCCC | 102 | NM_005944.5 | Neuronal |
| Neuropilin 1 (NRP1, CD304) | CD304 | ACGTGGAAGTCTTCGATGGAG | CACCATGTGTTTCGTAGTCAGA | 138 | NM_003873.5 | Neuronal |
| Myocyte enhancer factor 2C | MEF2C | tcgtggagacgttgagaaag | ttcttgttcaatgcggaatc | 82 | NM_001193347.1 | Neuronal |
| Paired box 3 | PAX3 | AGCTCGGCGGTGTTTTTATCA | CTGCACAGGATCTTGGAGACG | 148 | NM_000438.5 | Neuronal |
| Paired box 9 | PAX9 | GGAGGAGTGTTCGTGAACGG | CGGCTGATGTCACACGGTC | 98 | NM_006194.3 | Neuronal |
